# Neuromodulatory interventions for Alzheimer’s disease

**DOI:** 10.3389/fnagi.2026.1925914

**Published:** 2026-10-08

**Authors:** Ruofan Wang, Botao Zhu, Qingda Guo, Jun Steed Huang, Wandong Zhang

**Affiliations:** 1 School of Advanced Technology, Algonquin College, Ottawa, ON, Canada; 2 Department of Electrical and Computer Engineering, Western University, London, ON, Canada; 3 Shenzhen Top-Tech Industrial Co., Ltd., Shenzhen, Guangdong, China; 4 School of Information Technology, Carleton University, Ottawa, ON, Canada; 5 Northern Innovation on Quantum Technologies Inc., Ottawa, ON, Canada; 6 Faculty of Medicine, University of Ottawa, Ottawa, ON, Canada; 7 Human Health Therapeutics Research Center, National Research Council of Canada, Ottawa, ON, Canada

**Keywords:** Alzheimer’s disease, deep brain stimulation, focused ultrasound, gamma oscillations, mild cognitive impairment, neuromodulation, transcranial alternating current stimulation, transcranial direct current stimulation

## Abstract

Alzheimer’s disease (AD) and mild cognitive impairment (MCI) represent an escalating global public health challenge with major socioeconomic consequences. Although anti-amyloid monoclonal antibodies have introduced disease-modifying treatment options for early symptomatic AD, their benefits remain modest and must be balanced against cost, monitoring burden, and safety risks. Neuromodulatory interventions have therefore emerged as promising investigational strategies that may complement existing therapeutic approaches. This narrative review evaluates current clinical and neurophysiological evidence for seven major neuromodulation approaches: 40 Hz gamma rhythm sensory stimulation, also known as gamma entrainment using sensory stimulation (GENUS), transcranial alternating current stimulation (tACS), transcranial magnetic stimulation (TMS), transcranial direct current stimulation (tDCS), focused ultrasound (FUS), deep brain stimulation (DBS), and transcranial photobiomodulation (tPBM). Integrating clinical findings with mechanistic evidence, we examine synaptic plasticity, neuroinflammation, glymphatic clearance, oscillatory entrainment, and cholinergic signaling as candidate therapeutic mechanisms. A comparative matrix summarizes target specificity, invasiveness, stimulation parameters, and translational status. Finally, we discuss key barriers, including biological heterogeneity, protocol standardization, durability of benefit, caregiver burden, and the uncertain implications of continuous network stimulation for future personalized and closed-loop interventions.

## 1 Introduction

Over the last half-century, dramatic advances in medical science have significantly increased human life expectancy, without an equivalent increase in the “wellness span” or functional longevity of the aging population. By 2050, more than 130 million people worldwide are projected to be living with dementia (Prince et al., 2015). Alzheimer’s disease is the most common cause of dementia. Pathologically, AD is characterized by extracellular amyloid-β plaques, intracellular hyperphosphorylated tau aggregation, neuroinflammation, synaptic dysfunction, and neuronal loss (Scheltens et al., 2021). In the United States, an estimated 7.4 million adults aged 65 years or older are living with clinical Alzheimer’s dementia in 2026. Total health care, long-term care, and hospice payments for people aged 65 years or older with dementia are estimated at $409 billion in 2026 and are projected to approach $1 trillion by 2050 (Alzheimer’s Association, 2026). Crucially, epidemiological modeling suggests that introducing an intervention capable of delaying the clinical onset of AD by just 5 years would reduce the total number of cases by over 3 million and reduce overall healthcare costs by more than 33% by 2050 (Alzheimer’s Association, 2015).

In this context, targeting patients during high-risk transitional states, such as subjective cognitive decline (SCD) and mild cognitive impairment (MCI), represents an important window for early intervention. MCI involves objectively demonstrable cognitive deficits that do not yet severely impair activities of daily living, and affected individuals face a markedly increased risk of conversion to AD (Alzheimer’s Association, 2026). Similarly, approximately 15 million older Americans meet the clinical criteria for SCD, reporting cognitive worsening despite normal objective test performance; nearly 40% of them progress to dementia within 20 years (Buckley et al., 2016; Jessen et al., 2020; Slot et al., 2019; van Maurik et al., 2019).

Newer disease-modifying immunotherapies, including anti-amyloid monoclonal antibodies approved by the U.S. Food and Drug Administration, such as lecanemab and donanemab, have demonstrated statistically significant but clinically modest slowing of cognitive and functional decline in early symptomatic AD (Sims et al., 2023; van Dyck et al., 2023). Their use also requires careful patient selection, amyloid confirmation, magnetic resonance imaging (MRI) monitoring, and management of amyloid-related imaging abnormalities. This therapeutic gap highlights the need for cost-effective, accessible, and safe non-pharmacological interventions. Over the past decade, neuromodulatory interventions have emerged as promising investigational strategies for modifying disrupted neural activity and network function in AD. In this review, neuromodulation is used as an umbrella term for sensory, magnetic, electrical, acoustic, optical, and implanted stimulation approaches that influence neural activity, neural circuits, or brain network function.

The review examines recent advances in these approaches, with particular emphasis on their clinical evidence, neurophysiological mechanisms, therapeutic targets, and translational challenges. The literature-search and study-selection approach used for this narrative review is described in Section 2. This review considers AD not only as a molecular proteinopathy but also as a disorder involving circuit timing, network connectivity, and neuromodulatory function. This perspective helps explain the potential relevance of neuromodulation, as different modalities may restore disrupted oscillations, enhance residual neuronal plasticity, improve network communication, facilitate therapeutic delivery or clearance pathways, or support neuronal bioenergetics and neurovascular function.

## 2 Literature search and study selection

An updated structured literature search was conducted to improve the transparency and completeness of this narrative review. PubMed/MEDLINE, Web of Science Core Collection, and Scopus were searched from database inception through August 25, 2026. The search combined disease-related terms (“Alzheimer’s disease,” “Alzheimer disease” and “mild cognitive impairment”) with modality-specific terms, including “40 Hz gamma stimulation,” “gamma entrainment,” “GENUS,” “transcranial alternating current stimulation,” “tACS,” “transcranial magnetic stimulation,” “TMS,” “rTMS,” “transcranial direct current stimulation,” “tDCS,” “focused ultrasound,” “FUS,” “deep brain stimulation,” “DBS,” “transcranial photobiomodulation,” and “tPBM.” Additional searches combined the modality terms with mechanism-related keywords, including “synaptic plasticity,” “neuroinflammation,” “microglia,” “glymphatic clearance,” “cholinergic,” “amyloid,” “tau,” and “cerebral blood flow.” Reference lists of relevant reviews and eligible primary studies were also examined to identify additional publications. ClinicalTrials.gov was consulted for relevant ongoing or registered clinical studies.

Studies were considered eligible when they evaluated one of the seven neuromodulatory modalities reviewed in this article in patients with AD or MCI, or when they provided mechanistic evidence directly relevant to the biological rationale of these interventions. Priority was given to randomized controlled trials, sham-controlled studies, prospective clinical studies, systematic reviews and meta-analyses, mechanistic human studies, and clinically relevant registered trials. Preclinical studies were included selectively when they provided direct evidence regarding mechanisms such as oscillatory entrainment, synaptic plasticity, neuroinflammation, glymphatic clearance, or neuromodulatory circuit function. Preclinical findings were used to support biological plausibility rather than to infer clinical efficacy in AD. Studies were excluded when they were unrelated to AD or MCI, did not examine one of the reviewed modalities or a directly relevant mechanism, duplicated previously reported findings without providing additional information, or lacked sufficient methodological or outcome information for interpretation. Titles and abstracts were screened for relevance, followed by full-text assessment of potentially eligible studies, and final inclusion was based on the criteria above and relevance to the review objectives.

Randomized and sham-controlled clinical studies and meta-analyses were given greater weight when evaluating therapeutic efficacy. Mechanistic human studies were used primarily to interpret neurophysiological effects, whereas uncontrolled feasibility studies and preclinical experiments were interpreted more cautiously.

## 3 Neurophysiological basis of cognitive networks in Alzheimer’s disease

The execution of complex, goal-directed behaviors requires coordinated activation and functional synchronization of neurons across large-scale neural networks involving frontal, parietal, and limbic/paralimbic regions. Oscillatory activity within these networks spans theta (4–8 Hz), alpha (8–12 Hz), beta (13–30 Hz), and gamma (approximately 30–80 Hz) bands and contributes to information processing, sensory integration, attention, and working memory (Gratton, 2018; Niendam et al., 2012). During active cognitive engagement, task-relevant cortical regions often show transient increases in gamma-band synchronization, whereas lower-frequency activity may suppress or modulate task-irrelevant processing (Semprini et al., 2021). Gamma rhythms are broadly associated with large-scale brain network activity and cognitive phenomena. In the prefrontal cortex, particularly within the dorsolateral prefrontal cortex (DLPFC), localized gamma synchronization is a marker of working-memory maintenance and retrieval; this activity is driven by fast-spiking parvalbumin-positive GABAergic interneurons that coordinate rhythmic firing of excitatory pyramidal cells (Honkanen et al., 2015; Tallon-Baudry et al., 1998). Alzheimer’s disease is a multifactorial disorder in which amyloid-β (Aβ) and tau pathologies are associated with synaptic dysfunction, abnormal neuronal activity, and alterations in gamma-band oscillations (Traikapi and Konstantinou, 2021). AD is also increasingly recognized as a “disconnection syndrome” involving widespread disruption of large-scale networks, particularly the default mode network (DMN) and frontoparietal control systems (Menon, 2011; Scheltens et al., 2021). Alterations in high-frequency neural activity, including reduced gamma-band synchronization and abnormal task-related oscillatory responses, have been reported in patients with AD and MCI (Koenig et al., 2005; Kurimoto et al., 2012; Stam et al., 2002). The accumulation of soluble Aβ oligomers and hyperphosphorylated tau (p-tau) directly disrupts the intrinsic excitatory-inhibitory balance of neural circuits, and Aβ deposition selectively impairs parvalbumin-positive interneuron function, leading to a loss of gamma-band power, network desynchronization, and subsequent cognitive deterioration (Traikapi and Konstantinou, 2021). Reduced gamma activity and neuronal network desynchronization may therefore represent potential targets for neuromodulatory interventions aimed at restoring more physiological patterns of neural activity. Beyond gamma-band abnormalities, quantitative electroencephalography (EEG) studies have identified broader alterations in spectral and microstate dynamics in AD. Compared with frontotemporal dementia, AD has been associated with greater delta-band spectral power; while comparisons with healthy controls have shown reduced global field power and microstate occurrence together with prolonged microstate duration (Iqbal et al., 2026).

Alzheimer’s disease-related cognitive decline is also associated with progressive degeneration of the basal forebrain cholinergic system, particularly the nucleus basalis of Meynert (NBM). NBM cholinergic neurons provide widespread innervation to the neocortex and contribute to attention, memory-related cortical processing, and cortical plasticity (Mesulam, 2013). This cholinergic deficit can be non-invasively monitored in humans using transcranial magnetic stimulation (TMS) to assess short-latency afferent inhibition (SAI), an established neurophysiological index of central cholinergic transmission that is characteristically impaired in patients with AD (Di Lazzaro et al., 2002).

The below Section “4 Clinical and mechanistic evidence for neuromodulatory interventions” reviews seven neuromodulatory approaches for Alzheimer’s disease, including 40 Hz gamma rhythm sensory stimulation, transcranial alternating current stimulation (tACS), TMS, transcranial direct current stimulation (tDCS), focused ultrasound (FUS), deep brain stimulation (DBS), and transcranial photobiomodulation (tPBM). These modalities differ in their mechanisms of stimulation, anatomical targets, invasiveness, and principal biological effects. Their mechanistic and clinical evidence is examined individually within four functional classes before their therapeutic characteristics and translational status are compared across approaches.

## 4 Clinical and mechanistic evidence for neuromodulatory interventions

The seven neuromodulatory modalities reviewed in this section are organized into four functional classes according to their primary modes of intervention and principal neurophysiological effects: rhythm-based approaches, cortical excitability approaches, anatomically targeted approaches, and optical neuromodulation. The individual modalities are discussed within these four classes, which provide a consistent organizational framework for the modality-specific evidence, cross-modality comparison, and concluding synthesis presented in the subsequent sections. For each modality, mechanistic and preclinical findings are distinguished from human clinical evidence, with explicit consideration of evidence maturity and limitations.

### 4.1 Rhythm-based approaches

Rhythm-based approaches aim to modulate pathological neural oscillations and restore more physiological patterns of network synchronization. This category includes 40 Hz gamma sensory stimulation and tACS, which engage gamma-band activity through sensory and electrical stimulation, respectively.

#### 4.1.1 40 Hz gamma rhythm sensory stimulation

Non-invasive gamma entrainment using sensory stimulation (GENUS) uses rhythmic sensory stimulation at 40 Hz to entrain gamma oscillations through sensory pathways (Traikapi and Konstantinou, 2021). In AD-related mouse models, daily 40 Hz light and sound entrained gamma rhythms, reduced Aβ and p-tau pathologies, prevented neuronal loss, preserved synaptic density, and altered microglial morphology toward a more neuroprotective state (Adaikkan et al., 2019; Iaccarino et al., 2016; Martorell et al., 2019). Animal studies also indicate that 40 Hz sensory stimulation may facilitate glymphatic Aβ clearance mechanisms (Murdock et al., 2024).

Early clinical studies have begun translating these findings to human patients, but the clinical evidence remains preliminary and should be interpreted cautiously (Traikapi and Konstantinou, 2021). In an early-stage, randomized, placebo-controlled trial of mild probable AD patients, daily 1-h exposure to combined 40 Hz light and sound over 3 months was safe, well tolerated, and capable of entraining widespread cortical regions. Active GENUS participants also showed signals of reduced ventricular dilation, hippocampal-volume stabilization, enhanced functional connectivity within the DMN and medial visual networks, and improved performance on the face-name association delayed recall test (Chan et al., 2022). Similarly, a 6-months sham-controlled study by Cimenser et al. (2021) reported reduced nighttime restlessness and stabilization of daily functioning in AD patients, although sample size and sponsor involvement limit the strength of inference. On a structural level, Da et al. (2024) used MRI and diffusion tensor imaging to report attenuation of white-matter and myelin loss after 6 months of daily 40 Hz sensory stimulation. These imaging findings are promising but require independent replication and linkage to durable clinical outcomes. Substantial preclinical evidence supports the biological rationale for 40 Hz sensory stimulation, whereas human efficacy evidence remains preliminary.

#### 4.1.2 Transcranial alternating current stimulation

Transcranial alternating current stimulation applies low-intensity sinusoidal electrical currents to modulate endogenous neural oscillations in a frequency- and phase-specific manner (De Paolis et al., 2024). Unlike 40 Hz sensory stimulation, which induces gamma entrainment through visual, auditory, or tactile pathways, tACS applies an oscillatory electric field directly through scalp electrodes. In patients with AD and MCI, 40 Hz gamma-frequency tACS has been studied most extensively because it targets gamma-band abnormalities implicated in AD network dysfunction. Compared with sensory entrainment, tACS provides greater flexibility in electrode montage, network-node targeting, phase control, and potential integration with EEG-guided stimulation protocols (De Paolis et al., 2024). In a double-blind, sham-controlled, crossover trial, Benussi et al. (2022) randomized patients with early-stage AD to receive a single 60-min session of 40 Hz tACS over the precuneus. Active 40 Hz tACS improved episodic memory retrieval and normalized SAI, while exploratory predictor analysis associated apolipoprotein E (APOE) genotype with treatment response: the greatest cognitive improvement was observed in APOE ε4 non-carriers, and the magnitude of improvement progressively decreased in participants carrying one and two ε4 alleles (Benussi et al., 2022).

To evaluate repeated tACS sessions, Sprugnoli et al. (2021) conducted two open-label trials of daily 40 Hz tACS for 2 or 4 weeks targeting the temporal lobes. Perfusion-sensitive arterial spin labeling MRI showed a post-treatment increase in cerebral blood flow in the bilateral temporal lobes and hippocampus (Sprugnoli et al., 2021). More recently, Lu et al. (2025) reported that 15 consecutive days of 40 Hz tACS increased coupling between global blood oxygenation level-dependent signals and cerebrospinal fluid inflow, suggesting a possible effect on regional glymphatic flow that requires replication in larger controlled studies. To address the logistical barriers of frequent clinic-based treatments, Cappon et al. (2023) conducted a pilot feasibility study of home-based, remotely supervised 40 Hz tACS, demonstrating high adherence and acceptable safety. The memory findings are encouraging but remain exploratory because of the small sample size, open-label design, and need for caregiver-supported implementation. Current studies support short-term neurophysiological target engagement, but clinical efficacy and durability have not yet been established.

### 4.2 Cortical excitability approaches

Cortical excitability approaches seek to modulate neuronal excitability and enhance residual synaptic and network plasticity within cognitive and memory-related cortical regions. This category includes TMS and tDCS.

#### 4.2.1 Transcranial magnetic stimulation

Transcranial magnetic stimulation modulates cortical excitability through rapidly changing magnetic fields that induce electrical currents within cortical tissue (Pilato et al., 2012). Beyond its direct effects on cortical excitability, repetitive transcranial magnetic stimulation (rTMS) may influence neuroplastic and inflammatory processes relevant to neurodegenerative disease (Pilato et al., 2012). In a preclinical neuropathic-pain model, rTMS shifted microglial markers toward a less pro-inflammatory phenotype, with effects linked to inflammatory signaling pathways (Zhu et al., 2026). These findings provide indirect evidence that rTMS may influence neuroinflammatory mechanisms, although their relevance to AD remains largely preclinical.

A large meta-analysis by Pagali et al. (2024) reviewed 143 clinical studies on rTMS encompassing approximately 5,800 participants and reported statistically significant effects on global cognitive measures, including Mini-Mental State Examination (MMSE) [standardized mean difference (SMD) = 0.80], Montreal Cognitive Assessment (SMD = 0.85), and Alzheimer’s Disease Assessment Scale–Cognitive Subscale (ADAS-Cog) (SMD = −0.96), while also noting substantial protocol heterogeneity.

Regarding stimulation protocols, meta-analyses indicate that high-frequency (specifically 10–20 Hz) rTMS applied over multiple cortical sites, rather than single sites, yields the most robust and persistent cognitive enhancements (Pagali et al., 2024). The primary cortical targets investigated are the DLPFC (Ahmed et al., 2012) and the precuneus (Koch et al., 2022), the latter representing a primary hub of the DMN and a focal point of early AD pathology.

In a Phase 2, randomized, double-blind, sham-controlled trial, Koch et al. (2022) evaluated precuneus-targeted rTMS in patients with mild AD and reported slower cognitive decline with stabilization of DMN functional connectivity. Combining high-frequency rTMS with concurrent cognitive training may further enhance therapeutic engagement by activating task-relevant networks during stimulation (Sabbagh et al., 2020). However, the apparent synergy between stimulation and cognitive training should be regarded as hypothesis-generating until confirmed in larger multicenter trials with harmonized cognitive and biomarker endpoints. TMS has one of the broader human clinical evidence bases among the modalities reviewed here, although substantial protocol heterogeneity limits the certainty and generalizability of the reported cognitive benefits.

#### 4.2.2 Transcranial direct current stimulation

Transcranial direct current stimulation delivers weak, constant direct currents (typically 1–2 mA) through scalp electrodes to modulate resting membrane potentials, thereby altering cortical excitability and neuroplasticity in a polarity-dependent manner. Anodal tDCS depolarizes the neuronal membrane potential, increasing excitability and priming long-term potentiation-like plasticity, whereas cathodal tDCS hyperpolarizes the membrane, reducing excitability (Woods et al., 2016). The clinical utility of tDCS is supported by its low cost, portable design, and favorable safety profile, which make it suitable for remotely supervised, home-based applications (Im et al., 2019).

A recent systematic review and meta-analysis by Wang and Tian (2026) included 23 randomized controlled trials involving 823 patients with mild-to-moderate AD. tDCS was associated with improved global cognition (SMD = 0.66, 95% confidence interval 0.38–0.95; *p* < 0.01). Subgroup analyses suggested that significant memory improvement was observed in patients with more than 6 years of education and in protocols using 10 or fewer sessions, whereas executive-function effects were more evident after left DLPFC stimulation (Wang and Tian, 2026). Overall, the evidence favors anodal over cathodal stimulation, but the optimal montage, treatment duration, and maintenance schedule remain unresolved (Gangemi et al., 2021).

Despite encouraging short-term findings, evidence from longer-term and home-based tDCS trials in AD remains mixed. In a small randomized study of patients with early AD, Im et al. (2019) reported that 6 months of daily home-based prefrontal tDCS improved global cognition and language performance relative to sham stimulation and preserved regional cerebral glucose metabolism. More recently, a 26-weeks randomized, double-blind, sham-controlled trial in patients with mild AD found no significant benefit of active tDCS on global cognition, although a modest improvement in confrontation naming was observed (Yang et al., 2026). High-definition transcranial direct current stimulation (HD-tDCS) has also been evaluated specifically in patients with AD. In a pilot randomized, double-blind, sham-controlled trial involving 25 patients with Alzheimer’s dementia, LoBue et al. (2025) found no statistically significant between-group differences in cognitive outcomes after 10 sessions of medial prefrontal HD-tDCS, although moderate-to-large effect sizes were observed for verbal learning and phonemic fluency, with clinically meaningful improvement in a subset of participants. These findings indicate that the long-term efficacy of conventional tDCS and the potential cognitive benefits of HD-tDCS both require confirmation in larger controlled studies. Randomized clinical evidence for tDCS is relatively broad, but longer-term efficacy remains inconsistent.

### 4.3 Anatomically targeted approaches

Anatomically targeted approaches prioritize focal access to cortical or deep brain structures relevant to AD pathology and cognitive function. This category includes FUS and DBS, although the two modalities differ substantially in invasiveness and therapeutic mechanism: FUS provides non-invasive acoustic targeting and can also facilitate blood-brain barrier (BBB) opening, whereas DBS directly modulates deep neural circuits through implanted electrodes.

#### 4.3.1 Focused ultrasound

Focused ultrasound is a non-invasive, image-guided technology that delivers low-frequency acoustic energy through the intact skull to targeted deep brain structures with millimeter precision (Tyler et al., 2008). Among the approaches reviewed here, FUS has a distinctive dual role because it can directly modulate neural activity or transiently open the blood-brain barrier to facilitate therapeutic delivery. In patients with AD, a Phase 1 study established the safety and feasibility of MRI-guided FUS-mediated BBB opening in the frontal lobe (Lipsman et al., 2018), while FUS-mediated BBB opening combined with aducanumab accelerated regional Aβ plaque reduction compared with systemic antibody exposure alone (Aljehani et al., 2024; Rezai et al., 2024). This finding remains mechanistically important, although aducanumab has since been discontinued (Aljehani et al., 2024).

Beyond drug delivery, FUS-mediated BBB opening alone may influence pathological protein burden: Ye et al. (2025) evaluated repeated, large-volume bilateral frontal BBB opening and reported reductions in Aβ plaque levels together with improvements in neuropsychiatric symptoms. Furthermore, low-intensity FUS can be applied without microbubbles for direct neuromodulation, potentially altering neuronal membrane potentials and activating mechanosensitive ion channels (Tyler et al., 2008). Additionally, Beisteiner et al. (2020) introduced transcranial pulse stimulation, which uses ultrashort ultrasound pulses to stimulate distributed cortical regions and has shown preliminary cognitive benefit in patients with AD. Human evidence is strongest for the safety and feasibility of FUS-mediated BBB opening, whereas the therapeutic efficacy of direct FUS neuromodulation in AD remains investigational.

#### 4.3.2 Deep brain stimulation

Deep brain stimulation is an invasive surgical treatment that modulates neural activity through implanted depth electrodes in specific brain structures (Jiang et al., 2022; Laxton et al., 2010). In patients with AD and MCI, it has been investigated to restore dysfunctional memory circuits through low-frequency stimulation (20 Hz) of the NBM or high-frequency stimulation (130 Hz) of the fornix. Fornix DBS is intended to modulate the Papez circuit and hippocampal function. In a Phase I trial, bilateral fornix DBS in patients with mild AD was associated with reversal of regional glucose hypometabolism on positron emission tomography (PET) (Laxton et al., 2010). However, the Phase II ADvance trial did not demonstrate an overall cognitive benefit (Lozano et al., 2016). NBM DBS is intended to modulate basal forebrain cholinergic circuitry and associated cortical networks; in a study of eight patients with advanced AD, short-term MMSE improvement was not sustained at 12-months follow-up (Jiang et al., 2022). A comparative study of bilateral fornix versus NBM DBS in severe AD found both approaches safe, with greater improvement in neuropsychiatric symptoms after NBM DBS (Xu et al., 2025). Evidence for cognitive efficacy in AD remains limited and inconsistent and must be weighed against the procedural risks of invasive stimulation.

### 4.4 Optical neuromodulation

Optical neuromodulation uses light-based stimulation to influence cellular and neurovascular processes. In this review, this category centers on tPBM, which uses near-infrared light to modulate mitochondrial bioenergetics and cerebral blood flow.

#### 4.4.1 Transcranial photobiomodulation

Transcranial photobiomodulation is a non-invasive neuromodulation approach that delivers near-infrared light through the scalp and skull to superficial cortical regions. Absorption of these photons by cytochrome c oxidase is proposed to enhance electron transport and oxidative phosphorylation, while nitric oxide release from mitochondrial binding sites may facilitate vasodilation and regional cerebral blood flow. Together, these proposed mechanisms could support cellular bioenergetics and neurovascular function (Chung et al., 2012; Hamblin, 2024). In a randomized, double-blind, placebo-controlled trial of patients with MCI due to AD, Chun and colleagues evaluated self-administered 808 nm tPBM over the bilateral dorsolateral prefrontal cortex six times weekly for 12 weeks. Active treatment was safe and was associated with significantly improved cognitive performance; mean Korean-version Montreal Cognitive Assessment scores increased by 3.87 ± 2.51 points, compared with a 0.74 ± 2.85-point decline with placebo (*p* < 0.001) (Chun et al., 2026). These findings support further evaluation of tPBM as a non-pharmacological intervention for MCI due to AD, but its durability and potential to delay progression to dementia remain to be established. Clinical evidence for tPBM is encouraging but remains limited, while its mechanistic relevance to AD and the durability of clinical benefit require further confirmation.

Table 1 summarizes the seven modalities within the four functional classes and compares their target and invasiveness, principal mechanisms and stimulation parameters, and clinical evidence and maturity.

**TABLE 1:** Cross-modality comparison of neuromodulatory interventions for Alzheimer’s disease.

Modality	Target and invasiveness	Mechanism and typical parameters	Clinical evidence and maturity
40 Hz gamma sensory stimulation	Broad, non-invasive sensory-network entrainment; downstream hippocampal and cortical effects (Chan et al., 2022).	Visual/auditory 40 Hz stimulation, typically 1 h/day; proposed effects include gamma entrainment, microglial remodeling, and vascular/glymphatic mechanisms (Chan et al., 2022; Martorell et al., 2019; Murdock et al., 2024).	Early controlled human studies; efficacy remains preliminary and requires independent replication (Chan et al., 2022; Cimenser et al., 2021; Da et al., 2024).
Transcranial alternating current stimulation	Non-invasive rhythmic stimulation of network nodes such as precuneus or angular gyrus (Benussi et al., 2022; Cappon et al., 2023).	Sinusoidal current entrains endogenous oscillations; 40 Hz gamma protocols and combined gamma montages have been tested (Benussi et al., 2022; Cappon et al., 2023).	Small controlled/feasibility studies; target engagement shown, but efficacy and durability remain unconfirmed (Benussi et al., 2022; Cappon et al., 2023; Lu et al., 2025; Sprugnoli et al., 2021).
Transcranial magnetic stimulation	Non-invasive focal stimulation of cortical hubs such as DLPFC and precuneus (Koch et al., 2022; Pagali et al., 2024).	Magnetic induction of cortical currents; high-frequency 10–20 Hz and intermittent theta-burst stimulation protocols have been investigated, with substantial variation in stimulation intensity and treatment duration across studies (Pagali et al., 2024).	RCT and meta-analytic evidence; relatively broad human evidence base, but protocol heterogeneity limits generalizability (Koch et al., 2022; Pagali et al., 2024).
Transcranial direct current stimulation	Non-invasive cortical stimulation, commonly targeting prefrontal and other cognitive-network regions in AD (Im et al., 2019; Wang and Tian, 2026).	Weak direct current shifts resting membrane polarization; typical protocols use 1–2 mA for 20–30 min over DLPFC or temporoparietal targets (Wang and Tian, 2026; Woods et al., 2016).	Multiple RCTs/meta-analysis; short-term benefit reported, but long-term efficacy remains inconsistent (Im et al., 2019; LoBue et al., 2025; Wang and Tian, 2026; Yang et al., 2026).
Focused ultrasound	Non-invasive, image-guided targeting of cortical or deep brain structures (Lipsman et al., 2018; Tyler et al., 2008).	Pulsed ultrasound can transiently open the BBB in the presence of microbubbles or directly modulate neural activity at low intensity without microbubbles (Lipsman et al., 2018; Rezai et al., 2024; Tyler et al., 2008; Ye et al., 2025).	Early human evidence supports BBB-opening feasibility; direct neuromodulatory efficacy remains investigational (Beisteiner et al., 2020; Lipsman et al., 2018; Rezai et al., 2024; Ye et al., 2025).
Deep brain stimulation	Invasive implanted electrodes targeting deep memory and cholinergic structures (Jiang et al., 2022; Laxton et al., 2010).	Fornix stimulation has typically used high-frequency protocols (∼130 Hz), whereas NBM stimulation has used lower-frequency protocols (∼20 Hz); intermittent NBM-DBS is currently under clinical investigation (ClinicalTrials.gov., 2026; Jiang et al., 2022; Laxton et al., 2010).	Early-phase and Phase II studies; cognitive efficacy remains inconsistent and procedural risk is substantial (Jiang et al., 2022; Laxton et al., 2010; Lozano et al., 2016; Xu et al., 2025).
Transcranial photobiomodulation (tPBM)	Non-invasive near-infrared stimulation of superficial cortical regions (Chun et al., 2026; Chung et al., 2012; Hamblin, 2024).	Proposed effects include modulation of mitochondrial bioenergetics and neurovascular function; one clinical trial used 808 nm stimulation over the bilateral DLPFC six times weekly for 12 weeks (Chun et al., 2026; Chung et al., 2012; Hamblin, 2024).	Randomized evidence is encouraging but limited; independent replication and longer follow-up are needed (Chun et al., 2026).

## 5 Critical discussion and translational challenges

The comparative matrix highlights distinct translational profiles across the four functional classes introduced in Section “4 Clinical and mechanistic evidence for neuromodulatory interventions.” Rhythm-based approaches, including 40 Hz sensory stimulation and tACS, are most directly aligned with disrupted neural oscillations. Cortical excitability approaches, including TMS and tDCS, have accumulated broader non-invasive clinical experience but remain limited by variability in stimulation targets and protocols. Anatomically targeted approaches, including FUS and DBS, provide more focal access to therapeutically relevant brain regions, although they differ substantially in invasiveness and therapeutic mechanism: FUS can provide direct neuromodulation or facilitate therapeutic delivery through transient BBB opening, whereas DBS directly engages deep memory and cholinergic circuits through implanted electrodes. Optical neuromodulation with tPBM provides a non-invasive strategy targeting neuronal bioenergetics and neurovascular function, although its clinical evidence remains comparatively limited. Taken together, these differences highlight a central translational challenge. Future studies need to determine which patients, disease stages, and biological targets are best matched to each modality rather than treating neuromodulatory interventions as a homogeneous therapeutic category. Across these four functional classes, several cross-cutting challenges remain, including interindividual variability, parameter optimization, durability, and the risks associated with invasive or sustained network modulation.

The maturity of the evidence also differs substantially across modalities. TMS and tDCS have the broadest human clinical evidence base, including randomized trials and meta-analyses, although protocol heterogeneity and mixed longer-term findings limit certainty (Im et al., 2019; Koch et al., 2022; Pagali et al., 2024; Wang and Tian, 2026; Yang et al., 2026). Evidence for 40 Hz sensory stimulation and tACS is supported by early controlled human studies and target-engagement findings but remains less mature and requires larger independent trials (Benussi et al., 2022; Cappon et al., 2023; Chan et al., 2022; Cimenser et al., 2021; Da et al., 2024; Lu et al., 2025; Sprugnoli et al., 2021). For FUS, human evidence is strongest for the feasibility of BBB opening, while direct neuromodulatory efficacy remains investigational (Beisteiner et al., 2020; Lipsman et al., 2018; Rezai et al., 2024; Ye et al., 2025). DBS has early-phase and Phase II clinical evidence but has not shown consistent cognitive efficacy and carries substantial procedural burden (Jiang et al., 2022; Laxton et al., 2010; Lozano et al., 2016; Xu et al., 2025). tPBM has encouraging randomized clinical evidence, but the evidence base remains comparatively limited and requires independent replication (Chun et al., 2026).

### 5.1 Individual biological and genetic heterogeneity

In transcranial electrical stimulation, including tDCS and tACS, current distribution is influenced by individual differences in head and tissue morphology, including skull thickness and cortical anatomy (Krause and Cohen Kadosh, 2014). Age-related cortical atrophy and associated changes in cerebrospinal-fluid geometry may further alter electric-field distribution. Individualized MRI-guided computational head modeling may therefore help optimize electrode placement and stimulation intensity in future protocols.

Furthermore, biological and genetic factors may modify neurophysiological responsiveness (Krause and Cohen Kadosh, 2014). For instance, in an exploratory analysis of 40 Hz tACS in early AD, APOE ε4 non-carriers showed the greatest cognitive improvement, whereas the magnitude of improvement progressively decreased in participants carrying one and two ApoE ε4 alleles (Benussi et al., 2022).

### 5.2 Parameter optimization, dosage extrapolation, and durability

The durability of treatment effects remains uncertain across non-invasive neuromodulatory modalities, making maintenance schedules an important consideration in future trials. The feasibility of sustained home-based stimulation has been explored for modalities such as tACS (Cappon et al., 2023). However, long-term treatment also introduces practical and family-level considerations. In a recent 26-weeks home-based tDCS trial, active tDCS did not improve global cognition, domain-level effects were small and inconsistent, and caregiver-reported family quality of life declined (Yang et al., 2026). Future studies should evaluate approaches for predicting longer-term treatment response and identifying individualized maintenance schedules. Data-driven models may contribute to this effort, but their clinical utility will require prospective validation.

### 5.3 Invasive risks and network perturbation

For invasive neuromodulatory approaches such as DBS, the risks associated with neurosurgical implantation, including intracranial hemorrhage, infection, and lead displacement, must be carefully balanced against the expected clinical benefit, particularly in older and medically vulnerable patients with AD (Laxton et al., 2010). One emerging conceptual concern is that conventional continuous neuromodulation may act as a sustained network perturbation rather than an information-specific intervention, potentially affecting physiological as well as pathological network dynamics. Within this view, intermittent, adaptive, or closed-loop stimulation paradigms may better preserve physiologic information processing and network flexibility than fixed continuous stimulation protocols. However, this hypothesis remains largely theoretical and requires substantial experimental validation in both animal models and human clinical studies. These considerations have contributed to growing interest in intermittent and other non-continuous stimulation paradigms, including ongoing clinical investigation of intermittent NBM-DBS in AD (ClinicalTrials.gov., 2026).

## 6 Limitations and challenges in current evidence

Despite encouraging findings, several important limitations remain across the literature on neuromodulatory interventions for Alzheimer’s disease. Many studies are limited by small sample sizes, different inclusion and exclusion criteria, heterogeneous stimulation protocols, short follow-up durations, inconsistent sham-control methodologies, and variable outcome measures. Inter-individual variability in responsiveness is substantial and may reflect differences in cortical atrophy, disease stage, baseline network integrity, medication exposure, APOE genotype, and targeting accuracy. Reproducibility across centers remains limited, particularly for tDCS, tACS, and emerging ultrasound protocols. Larger multicenter randomized controlled trials with standardized protocols, biomarker-guided patient stratification, and longer durability assessments will therefore be necessary before routine clinical adoption can be justified.

Future studies should also separate symptomatic cognitive enhancement from disease modification. Trials should include prespecified primary endpoints, adequate sham blinding checks, adverse-event reporting, caregiver-burden measures, and biomarkers such as amyloid/tau PET, structural MRI, EEG or magnetoencephalography oscillatory markers, or functional-connectivity outcomes. Without these safeguards, short-term improvements on cognitive scales may overestimate clinically meaningful benefit. Potentially combination of anti-amyloid antibody therapies with neuromodulation may be explored in future.

## 7 Conclusion and future perspectives

Current clinical and neurophysiological evidence supports neuromodulatory interventions as promising but still investigational therapeutic approaches for MCI and Alzheimer’s disease. As with other emerging treatment strategies for MCI and AD, their clinical therapeutic effects remain to be defined and may ultimately vary across modalities, disease stages, and patient subgroups. Depending on the strength of future evidence, neuromodulation may be used in combination with pharmacological and non-pharmacological interventions or, in selected contexts, as a therapeutic strategy in its own right. Four integrated conclusions emerge from the four functional classes considered in this review:

- First, rhythm-based approaches, including 40 Hz sensory stimulation and tACS, have progressed from basic laboratory science to early and mid-stage clinical testing, with converging evidence that rhythmic stimulation can entrain cortical activity and may influence microglial, vascular, and glymphatic pathways related to Aβ and p-tau pathology (Lu et al., 2025; Martorell et al., 2019; Murdock et al., 2024; Traikapi and Konstantinou, 2021).
- Second, cortical excitability approaches, including TMS and tDCS, have the broadest non-invasive clinical experience, but they require better harmonization of target selection, dose, maintenance schedules, and sham procedures before efficacy can be generalized across centers (Im et al., 2019; Pagali et al., 2024; Wang and Tian, 2026).
- Third, anatomically targeted approaches, including FUS and DBS, provide focal access to therapeutically relevant brain regions but occupy distinct translational positions. FUS can function as a non-invasive neuromodulatory tool and as a platform for transient BBB opening and therapeutic delivery, whereas DBS directly engages deep memory and cholinergic circuits at the cost of substantially greater procedural risk (Jiang et al., 2022; Laxton et al., 2010; Rezai et al., 2024; Xu et al., 2025; Ye et al., 2025).
- Fourth, optical neuromodulation with tPBM represents an emerging non-invasive strategy that may influence mitochondrial bioenergetics, cerebral blood flow, and neurovascular function. Early clinical findings in MCI due to AD are encouraging, but the evidence base remains limited and requires confirmation in larger independent trials with longer follow-up (Chun et al., 2026; Chung et al., 2012; Hamblin, 2024).

Overall, the neuromodulatory approaches reviewed here represent a diverse family of mechanism-informed therapeutic strategies whose clinical roles are still evolving. Their future value may lie in modality-specific use, integration with pharmacological and non-pharmacological treatments, or application in selected patient groups where specific neural or network abnormalities can be targeted effectively. The next phase of the field should move from proof-of-concept stimulation toward precision protocols that match modality, target, frequency, dose, and maintenance schedule to disease stages and individual neurophysiology. Progress will depend on rigorously designed trials, individualized targeting, harmonized outcome measures, realistic assessment of caregiver burden, and transparent reporting of both positive and negative results.
